# Hypoxia-mediated impaired erythrocyte Lands’ Cycle is pathogenic for sickle cell disease

**DOI:** 10.1038/srep29637

**Published:** 2016-07-20

**Authors:** Hongyu Wu, Mikhail Bogdanov, Yujin Zhang, Kaiqi Sun, Shushan Zhao, Anren Song, Renna Luo, Nicholas F. Parchim, Hong Liu, Aji Huang, Morayo G. Adebiyi, Jianping Jin, Danny C. Alexander, Michael V. Milburn, Modupe Idowu, Harinder S. Juneja, Rodney E. Kellems, William Dowhan, Yang Xia

**Affiliations:** 1 Department of Biochemistry and Molecular Biology, University of Texas-Medical School, Houston, TX, USA; 2 Graduate School of Biomedical Science, University of Texas, Houston, TX, USA; 3 Metabolon, Inc., Durham, NC, USA; 4 Department of Internal Medicine, University of Texas-Medical School, Houston, TX, USA

## Abstract

Although Lands’ cycle was discovered in 1958, its function and cellular regulation in membrane homeostasis under physiological and pathological conditions remain largely unknown. Nonbiased high throughput metabolomic profiling revealed that Lands’ cycle was impaired leading to significantly elevated erythrocyte membrane lysophosphatidylcholine (LysoPC) content and circulating and erythrocyte arachidonic acid (AA) in mice with sickle cell disease (SCD), a prevalent hemolytic genetic disorder. Correcting imbalanced Lands’ cycle by knockdown of phospholipase 2 (cPLA2) or overexpression of lysophosphatidycholine acyltransferase 1 (LPCAT1), two key enzymes of Lands’ cycle in hematopoietic stem cells, reduced elevated erythrocyte membrane LysoPC content and circulating AA levels and attenuated sickling, inflammation and tissue damage in SCD chimeras. Human translational studies validated SCD mouse findings and further demonstrated that imbalanced Lands’ cycle induced LysoPC production directly promotes sickling in cultured mouse and human SCD erythrocytes. Mechanistically, we revealed that hypoxia-mediated ERK activation underlies imbalanced Lands’ cycle by preferentially inducing the activity of PLA2 but not LPCAT in human and mouse SCD erythrocytes. Overall, our studies have identified a pathological role of imbalanced Lands’ cycle in SCD erythrocytes, novel molecular basis regulating Lands’ cycle and therapeutic opportunities for the disease.

Cellular membranes from all of organisms consist of a bipolar lipid bilayer, which contains phospholipids (PLs), cholesterol, and proteins. PLs are major components of cellular membranes and play multiple important structural and cellular functions. PLs are synthesized by the Kennedy pathway, a *de novo* pathway in the Golgi and endoplasmic reticulum, and repaired by Lands’ cycle, a remodeling pathway^1^. Red blood cells (RBCs) are unique compared to other cells, they do not have *de novo* synthesis of PLs due to lack of Golgi and endoplasmic reticulum. As such, membrane maintenance and renewal depend solely on a functional Lands’ cycle, which is achieved by two concerted enzymes: phospholipases A_2_ (PLA2s) and lysophospholipid (LysoPL) acyltransferases (LPLATs). In the Lands’ cycle, PLA2s specifically hydrolyze the sn-2 position ester bond of phospholipids, which results in the formation of lysophospholipid. Subsequently, LPLATs as lipid repair enzymes transfer an acyl-group from acyl-CoA to lysophospholipid to regenerate phospholipids, completing the de-acylation/re-acylation repair cycle^2^. Although Lands’ cycle was discovered nearly 60 years ago and its speculated function is to modify fatty acid composition of PLs derived from the Kennedy pathway, its function and regulation in membrane homeostasis under physiological and pathological condition have remained poorly understood^1^.

Sickle cell disease (SCD) is the most prevalent hereditary hemolytic disorder caused by a single point mutation in the β–globin gene. Under chronic state, deoxygenated hemoglobin S (HbS) forms insoluble polymers and causes characteristic sickled erythrocyte morphology and promotes intravascular hemolysis. Moreover, one of the principal causes of hospitalization of SCD patients is acute vaso-occlusive crisis (VOC). VOC is the most dangerous condition because hypoxia promotes profound sickling and intravascular hemolysis^3^. Without interference, it rapidly progresses to a severe inflammatory response, vaso-occlusion, multiple organ damage and early death. Although it is well accepted that deoxygenation and polymerization of deoxygenated HbS are initial triggers for sickling, abnormal membrane lipid organization and composition was reported in sickled erythrocytes over three decades ago^4^,^5^,^6^,^7^. Early studies showed that abnormal membrane lipid composition is associated with increased intracellular calcium^8^, increased binding of hemoglobin^9^, enhanced flip-flop of PC and the exposure of PS on the outer leaflet^6^ and enhanced susceptibility of sickled erythrocytes to lipid peroxidation^10^. However, overall membrane specific lipid alteration in sickle erythrocytes, its *in vivo* pathological role and the mechanism causing changes of sickle erythrocyte membrane lipid composition are undetermined.

Here using nonbiased high throughput metabolomic profiling, we found a substantial increase in the concentration of LysoPLs in erythrocytes and AA in the circulation of SCD mice. These findings immediately suggest that Lands’ cycle in SCD erythrocytes is impaired. Extending from metabolomic screening, we conducted both mouse and human studies to systemically address a central question of role and mechanisms of alterations of PLs in SCD with a goal to identify pathogenic alterations in Lands’ cycle in this hemolytic disorder.

## Results

### Metabolomic screening and biochemical analysis reveal that erythrocyte lysophosphatidylcholine and circulating arahcidonic acid levels were most elevated together with impaired erythrocyte Lands’ cycle in SCD mice

**Figure 1:**
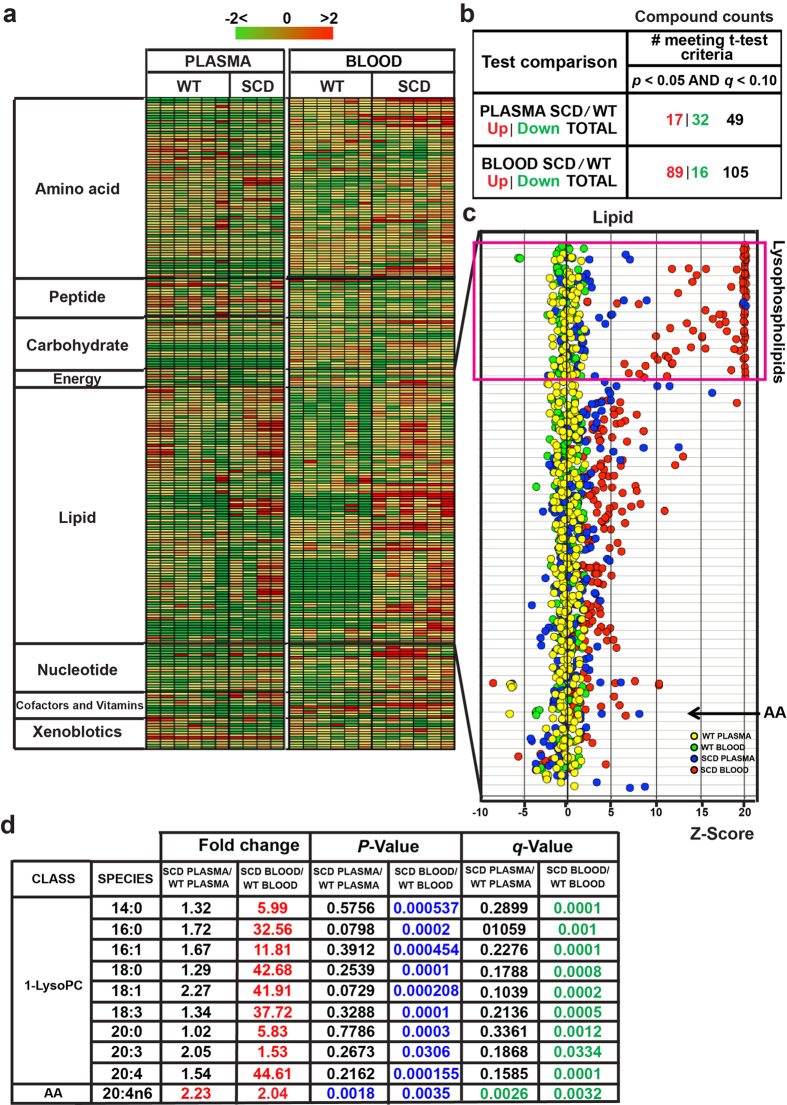
Metabolomic screening of mouse whole blood and plasma in WT and SCD mice. (**a**) Heat map showing the alteration of metabolite concentration of 8 groups (including amino acids, carbohydeates, cofactors, TCA cycle, lipids, nucleosides and metabolites, peptides and xenobiotics) shades of green and red represent an increase and decrease in metabolite concentration respectively; relative to the median metabolite level (see color scale). (**b**) Summary of significant altered metabolites in SCD mice compared to WT mice. (**c**) Z-score quantification of lipids detected in whole blood and plasma of both WT and SCD Tg mice (*n* = 4–6). Among all lipids detected, lysophospholipids were the most significantly elevated in whole blood of SCD Tg mice compared with that in WT mice (*n* = 6). AA was elevated in both plasma and whole blood of SCD Tg mice (*n* = 4–6). (**d**) Profiling of Lysophospholipids and AA detected in whole blood and plasma of both control and SCD Tg mice (*n* = 4–6). q-value is a measure of false discovery rate.

In an effort to precisely determine the overall erythrocyte and circulating lipid alteration in SCD, we conducted nonbiased high throughput metabolomic screening in the whole blood and plasma of controls and Berkeley SCD mice, a well-accepted humanized mouse model for SCD with sickling at steady state^11^,^12^. Metabolomic profiling successfully identified 251 metabolites in whole blood and plasma including eight categories such as amino acids, lipids and carbohydrates, etc (Fig. 1a). In whole blood, a total of 105 metabolites were significantly altered including 89 metabolites up and 16 metabolites down in SCD mice compared to the controls (*P* < 0.05, Fig. 1b). In the plasma, a total of 49 metabolites were significantly different including 17 metabolites up and 32 metabolites down in SCD mice compared to the controls (*P* < 0.05, Fig. 1b). Notably, Z-score analysis revealed that overall lipids in whole blood and plasma were most elevated (Fig. 1c). Specifically, LysoPLs including lysophosphatidylcholine (LysoPC), lysophosphatidylethalanomine (LysoPE) and lysophosphatidylserine (LysoPS) were significantly elevated in whole blood but not in the plasma of SCD mice (Fig. 1c). Among all of the elevated LysoPLs, LysoPC was elevated the most significantly (Fig. 1d). Moreover, most of the free fatty acids were also substantially elevated in whole blood but not in the plasma of SCD mice (Fig. 1c). Notably, arachidonic acid (AA) was increased significantly in both plasma and whole blood of SCD mice (Fig. 1c,d). Overall, nonbiased metabolomic screening revealed that the levels of LysoPLs, especially LysoPC, were most elevated in whole blood but not the plasma, while both plasma and erythrocyte AA levels were increased in SCD Tg mice (Fig. 1c,d).

**Figure 2:**
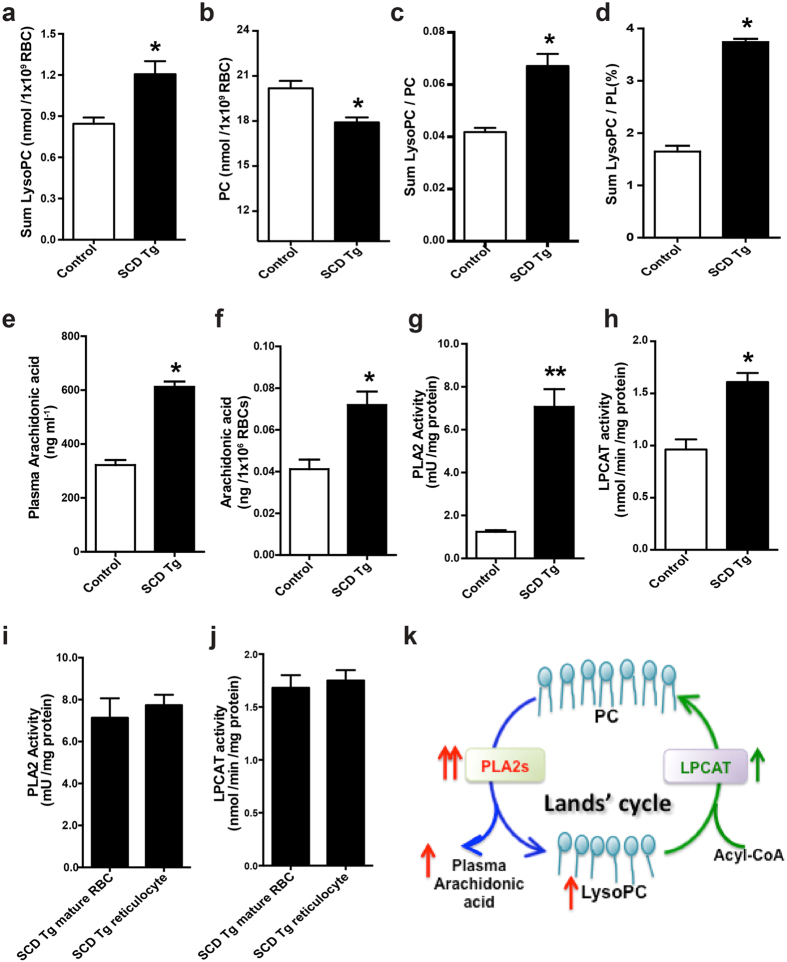
Biochemical analyses revealed increased lysophosphatidylcholine levels in the erythrocytes and increased arachidonic acid in plasma and erythrocytes of SCD mice due to imbalanced Lands’ cycle. (**a–d**) LysoPC (**a**), PC level (**b**), the ratio of LysoPC/PC (**c**) and the percentage of LysoPC/PL (**d**) in erythrocyte membrane preparations from control and SCD Tg mice. Error bars, SEM; n = 6 per group. (**e,f**) Plasma and erythrocyte AA levels were significantly increased in SCD Tg mice compared to that of controls. Error bars, SEM; n = 6 per group. (**g**) PLA2 activity was measured in the erythrocytes from control and SCD Tg mice. Error bars, SEM; n = 10 per group *P < 0.05 versus controls. **P < 0.01 versus controls. (**h**) LPCAT activity was detected in the erythrocytes from control and SCD Tg mice. Error bars, SEM; n = 8 per group. (**i,j**) PLA2 activity (**i**) and LPCAT activity (**j**) were measured in purified mature erythrocytes and reticulocytes from SCD Tg mice, respectively. Data are expressed as Mean ± SEM; n = 5 in each group. (**k**) Imbalanced Lands’ cycle favored the production of LysoPC generation from PC due to overly active PLA2s compared to LPCAT activity in SCD erythrocytes. PLA2s hydrolyze PC to release LysoPC and AA, while LPCAT catalyzes the reacylation at the *sn* -2 position of LysoPC into PC by using acyl-CoA.

We found that LysoPC levels were most elevated among all of the detected LysoPLs in whole blood of SCD mice and there was no difference of LysoPC in the plasma between the control and SCD mice (Fig. 1c,d). Because erythrocyte is the most abundant circulating cell type, it is possible that erythrocyte LysoPC levels are elevated in SCD mice as we seen in whole blood. To test our hypothesis, we chose to accurately measure erythrocyte membrane total LysoPC, phosphatidylcholine (PC) and PLs. First, we confirmed the results of metabolomic screen showing that total LysoPC content was significantly elevated in erythrocytes isolated from SCD Tg mice compared to the controls (Fig. 2a,b). LysoPC is generated from PC in the erythrocytes. As expected, we found that PC content in the SCD erythrocytes was significantly reduced (Fig. 2c). As such, the ratio of LysoPC/PC and the percentage of LysoPC to the total PLs (PL) (characterized by LysoPC/PL) were significantly increased in the erythrocytes isolated from SCD mice compared to the controls (Fig. 2d). Thus, metabolomics screening led us to discover that total membrane LysoPC levels, LysoPC/PC and LysoPC/PL ratio were significantly elevated, while total membrane PC levels were reduced in sickle mouse erythrocytes. Extending from quantification of membrane LysoPC, PC and PLs, we further validated our metabolomic screening showing that erythrocyte and plasma AA were significantly elevated to a similar level in SCD mice, implicating that erythrocytes are likely a major source for increased circulating AA seen in SCD mice (Fig. 2e,f).

Our findings showing increased membrane LysoPC content and reduced PC level in SCD erythrocytes and increased erythrocyte and circulating AA immediately raise the novel but compelling hypothesis that Lands’ cycle in sickle erythrocytes was imbalanced. To test this intriguing possibility, we conducted biochemical assays to quantify specific PLA2 activity and LPCAT activity. As expected, we found that erythrocyte PLA2 activity was significantly increased (over 6 fold) in SCD Tg mice compared to the control mice (Fig. 2f). However, erythrocyte LPCAT activity was increased only 1.7 fold in SCD Tg mice (Fig. 2g). To further distinguish whether elevated PLA2 and LPCAT activity seen in SCD mouse erythrocytes are due to increased reticulocytes in SCD Tg mice, we compared both enzyme activities in purified mature erythrocytes and reticulocytes from SCD Tg mice. We found that for each enzyme the activity in reticulocytes and erythrocytes was similar (Fig. 2h,i). Thus, these findings demonstrated that PLA2 and LPCAT activity were elevated to similar levels in erythrocytes and reticulocytes in SCD mice and were therefore independent of the age of the cells. Overall, we demonstrated that erythrocyte PLA2 activity is overly activated compared to LPCAT activity, repairing enzyme of Lands’ cycle in SCD mice, and thereby resulting in increased erythrocyte LysoPC and circulating and erythrocyte AA in SCD mice (Fig. 2j).

### Cytosolic PLA2 and LPCAT1 are the major components of Lands’ cycle functioning in mouse sickle erythrocytes

PLA2 comprises five main subtypes including secreted PLA2 (sPLA2), cytosolic PLA2 (cPLA2), calcium-independent PLA2 (iPLA2), platelet activating factor acetylhydrolases (PAF-AHs)^13^,^14^, and lysosomal PLA2 (LPLA2)^15^. In erythrocytes, three PLA2s, including sPLA2, cPLA2 and iPLA2, have been reported^16^,^17^. To determine which PLA2 is the major type functioning in mouse sickle erythrocytes, we chose to treat primary mouse sickle erythrocytes with methyl arachidonyl fluorophosphonate (MAFP)^18^,^19^,^20^,^21^ or pyrrophenone^22^, two most commonly used cPLA2s inhibitors, or BEL, a potent and specific iPLA2 inhibitor^23^,^24^. We found that MAFP and pyrrophenone significantly inhibited PLA2 activity in incubated mouse sickle erythrocytes in a dosage-dependent manner (Supplementary Fig. 1a,b). Of note, MAFP showed much more potent inhibitory effects on PLA2 activity compared to pyrrophenone. In contrast, BEL showed no obvious inhibitory effect on PLA2 activity at concentrations up to 40 μM (Supplementary Fig. 1c). Thus, these results indicated that cPLA2 is the main type of PLA2 functioning in mouse sickle erythrocytes.

Three members of the LPCAT family, encoded by three *AYTL* genes including *AYTL*-1, *AYTL*-2 and *AGPAT7*, were expressed in an erythroleukemic cell line^25^,^26^. In contrast to *Aytl*-1 and *Aytl*-3, the murine *Aytl*-2 gene was the only *Aytl* gene expressed in the erythroid lineage and its product was characterized as the lysophosphatidycholine reacylating enzyme in RBCs^26^. It was reported that the product of *Aytl*-2 gene was LPCAT1 in mouse lung tissue^27^. Therefore, we firstly detected LPCAT1 protein in the membrane of mature erythrocytes and reticulocytes isolated from SCD Tg mice by western blot. We found that LPCAT1 was highly expressed and there was no significant difference between these two cell types (Supplementary Fig. 1d). Functionally, we found that LPCAT activity in SCD mouse erythrocytes was significantly inhibited in a dosage-dependent manner by 2,2-dimethyl-N-(2,4,6-trimethoxyphenyl) dodecanamide (CI-976), one of the most commonly used specific LPCAT inhibitors^28^,^29^ (Supplementary Fig. 1e). Thus, the results of functional activity and protein expression led us to conclude that cPLA2 and LPCAT1 are two major components of Lands’ cycle functioning in sickle mouse erythrocytes.

### Knockdown of cPLA2 in bone marrow-derived cells attenuates sickling, hemolysis, inflammation and tissue damage by reducing erythrocyte LysoPC and circulating AA in SCD chimeras

**Figure 3:**
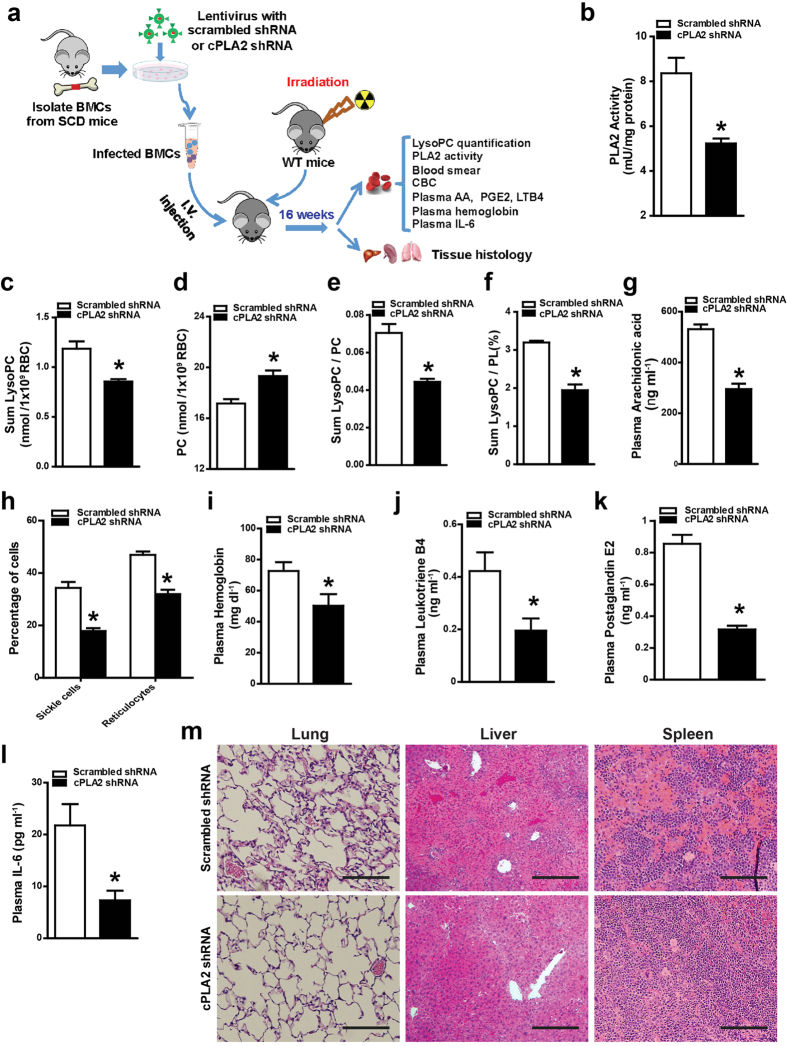
shRNA specific knockdown of cPLA2 in HSCs of BMT SCD chimeras attenuated sickling, hemolysis and inflammation. (**a**) Schematic flow of mouse treatment strategy and experimental procedure. (**b–f**) PLA2 activity (**b**), LysoPC content (**c**), PC content (**d**), the ratio of LysoPC/PC (**e**) and the percentage of LysoPC/PL (**f**) were quantified in the erythrocytes from SCD chimeras mice with scrambled shRNA or cPLA2 shRNA. Error bars, SEM; n = 5–7 per group. (**g**) Circulating AA was significantly decreased in SCD chimeras with specific cPLA2 knockdown. (**h**) Percentages of sickled cells and reticulocytes were significantly reduced in the SCD chimeras with HSC-specific cPLA2 knockdown. (**i–l**) cPLA2 knockdown in HSCs of BMT SCD chimeras decreased plasma hemoglobin levels (**i**), leukotriene B4 (**j**), prostaglandin E2 (**k**) and reduced circulating IL-6 (**l**). Values shown represent the mean ± SEM (*n* = 5–7). (**m**) H&E staining of spleens, livers, and lungs of SCD chimeras with HSC-specific cPLA2 knockdown and controls. *P < 0.05 versus SCD chimeras with BMCs infected with recombinant lentivirus encoding scrambled shRNA.

Erythrocytes are derived from hematopoietic stem cells (HSCs) in the bone marrow (BM) and they are the major circulating cells derived from BM. Thus, to determine the role of imbalanced Lands’ cycle due to overly induced erythrocyte cPLA2 activity-mediated increased erythrocyte LysoPC and circulating AA in SCD, we used lentiviral vectors encoding shRNA to specifically knockdown cPLA2 in HSCs of bone marrow cells (BMCs) isolated from SCD mice coupled with bone marrow transplantation (BMT). As illustrated in Fig. 3a, we transfected BMCs isolated from SCD mice infected with recombinant lentivirus encoding shRNAs specific for cPLA2 or scrambled shRNA. We determined approximately 75% of BMCs expressed shRNA viral vector encoding EGFP by using flow cytometry as described^30^. Following successful viral transduction, we transplanted the genetically modified BMCs from SCD mice to lethally irradiated WT recipients to generate SCD chimeras. Sixteen weeks after BMT, mice with specific cPLA2 shRNA knockdown or control shRNA with more than 95% HbS (Supplementary Fig. 2a) were used for further study including accurately measuring lipid content and the analysis of sickling, inflammation and tissue damage (Illustration in Fig. 3a). First, we found that erythrocyte PLA2 activity was significantly reduced in SCD chimeras with the specific cPLA2 shRNA knockdown compared to SCD control chimeras (Fig. 3b). Next, we found that decreased cPLA2 activity, resulting from the specific knockdown of cPLA2, led to a significant reduction of LysoPC and in turn increased PC level in the erythrocytes of SCD chimeras compared to SCD control chimeras (Fig. 3c,d). As such, both the ratio of LysoPC/PC and LysoPC/PL were significantly decreased in erythrocytes of SCD chimeras with the specific knockdown of cPLA2 compared to those of the SCD control chimeras (Fig. 3e,f). Similar to reduction of erythrocyte LysoPC, we found that circulating AA, another product generated by cPLA2, was also significantly reduced by knockdown of cPLA2 in BM-derived cells of SCD chimeras (Fig. 3g). Thus, we concluded that elevated cPLA2 in erythrocytes is a key factor contributing to imbalanced Lands’ cycle in SCD erythrocytes and underlying increased erythrocyte LysoPC and circulating AA in SCD mice.

Because we found that elevation of cPLA2 contributes to increased erythrocyte LysoPC, it is possible that increased membrane LysoPC plays a role in sickling, the central pathogenic process of SCD. As expected, we found that the percentages of sickled cells and reticulocytes in the SCD chimeras with specific knockdown of cPLA2 in hematopoietic cells were significantly reduced compared to those of the SCD chimera controls (Fig. 3h). Furthermore, we found that lentiviral knockdown of cPLA2 in BMCs of SCD chimeras led to significantly decreased hemolysis, as demonstrated by decreased plasma Hemoglobin (Fig. 3i). Because of the anti-sickling and anti-hemolytic effects, CBC analysis showed that knockdown of cPLA2 in SCD chimeras significantly increased the total number of erythrocytes, Hb concentration, and hematocrit (Supplementary Table 1). Additionally erythrocyte distribution width was significantly reduced in SCD chimeras with specific cPLA2 knockdown (Supplementary Table 1). Consistently, mean cellular Hb was also improved in SCD Tg mice by knockdown of cPLA2 (Supplementary Table 1). Thus, these quasi-genetic studies using cPLA2 knockdown in BMT SCD chimeras provide strong evidence that elevated cPLA2 activity contributes to sickling and subsequent hemolysis likely by causing LysoPC production in erythrocytes of SCD mice.

Because knockdown of cPLA2 in BMCs also led to decreased circulating AA, a precursor for inflammatory mediators including leukotriene and prostaglandin^31^, in SCD chimeras, it is possible that elevated cPLA2 also contributes to inflammation, a major complication in SCD. Supporting our hypothesis, CBC analysis showed that knockdown of cPLA2 significantly reduced the total number of white blood cells (Supplementary Table 1). Moreover, we found that circulating leukotriene B4, prostaglandin E2 and IL-6 levels were significantly decreased in the SCD chimeras with specific knockdown of cPLA2 (Fig. 3j–l). Thus, we demonstrated that knockdown of BM-derived cell cPLA2 leads to decreased erythrocyte cPLA2 activity and in turn reduces circulating AA and chronic inflammation.

Finally, we performed histological studies to determine the effects of knockdown of cPLA2 in BMCs in peripheral tissue damage in SCD chimeras. We found the specific knockdown of cPLA2 in SCD chimeras by shRNA in BMCs prevented the congestion, vascular damage and necrosis in multiple tissues, including lungs, livers, and spleens (Fig. 3m). Semiquantitative analysis of histological changes demonstrated that knockdown of cPLA2 in BMCs in SCD chimeras resulted in significant improvement in comparison with that in controls (Supplementary Fig. 2). Altogether, specific knockdown of cPLA2 in BMCs of SCD mice led us to conclude that elevated erythrocyte cPLA2 is a pathogenic factor to induce sickling, inflammation and tissue damage by inducing erythrocyte LysoPC production and circulating AA levels.

### Overexpression of LPCAT in bone marrow-derived cells attenuates sickling, hemolysis, inflammation and tissue damage by reducing LysoPC in erythrocytes and circulating arachidonic acid in SCD chimeras

**Figure 4:**
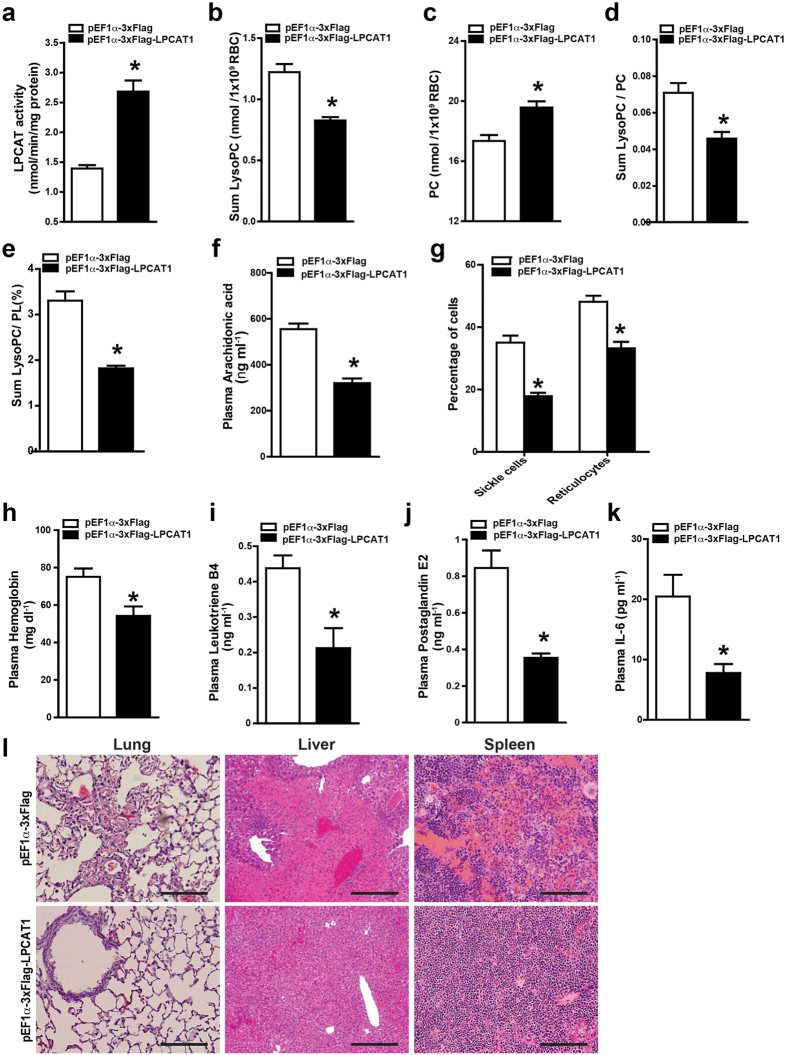
Overexpression of LPCAT1 in HSCs of BMT SCD chimeras attenuates sickling, hemolysis, inflammation and tissue damage. (**a–e**) LPCAT activity (**a**), LysoPC content (**b**), PC content (**c**), the ratio of LysoPC/PC (**d**) and the percentage of LysoPC/PL (**e**) were quantified in the erythrocytes from SCD chimeras with overexpression of LPCAT1 or control vector. Error bars, SEM; n = 6 per group. (**f**) Overexpression of LPCAT1 in HSCs of BMT SCD chimeras significantly decreased plasma AA levels. (**g**) Percentages of sickle cells and reticulocytes were significantly reduced in the SCD chimeras overexpressing LPCAT1 in HSCs. (**h–k**) Overexpression of LPCAT1 in HSCs decreased plasma hemoglobin (**h**), leukotriene B4 (**i**), prostaglandin E2 (**j**) and IL-6 (**k**) levels in SCD chimeras. Values shown represent the mean ± SEM (*n* = 6). (**l**) Hematoxylin and eosin stain shows histological changes in spleen, liver, and lung tissues of SCD chimeras 16 weeks after BMT. Scale bar, 10 μm. Values shown represent the mean ± SEM, n = 6 per group. *P < 0.05 versus SCD chimeras with BMCs infected with recombinant lentivirus packaging control vector.

Although LPCAT activity was elevated in SCD Tg mice compared with control mice (Fig. 2h), it did not increase as high as PLA2 in SCD mouse erythrocytes. Thus, to test the possibility that overexpression of LPCAT1 in HSC of BMT SCD chimeras can correct elevated LysoPC and circulating AA and sequential pathologic process of SCD, we overexpressed LPCAT1 using lentiviral vector in BMCs and conducted BMT. Sixteen weeks after BMT, mice with specific overexpression of LPCAT1 in BMCs or control lentivirus vetor with more than 95% HbS (Supplementary Fig. 3a) were used for further study including accurately measuring lipid content and systemic analysis of sickling, inflammation and tissue damage (Illustration in Fig. 3a). Supporting successful overexpression of LPCAT1 in bone marrow derived cells of SCD chimeras, we found that LPCAT activity was significantly increased in erythrocytes of SCD chimeras with the overexpression of LPCAT1 compared to those of SCD control chimeras (Fig. 4a). Similar to knockdown of PLA2 in BMCs in SCD chimeras, overexpression of LPCAT1 in erythrocytes of SCD chimeras significantly decreased erythrocyte membrane LysoPC levels (Fig. 4b) and increased PC levels (Fig. 4c). As such, the ratio of LysoPC/PC (Fig. 4d), the percentage of LysoPC/PL (Fig. 4e) and circulating AA (Fig. 4f) were significantly increased in SCD chimeras with overexpression of LPCAT1 in BMCs compared to SCD control chimeras. As such, the percentage of sickled erythrocytes and reticulocytes was significantly decreased in SCD chimeras with LPCAT1 overexpression compared to SCD control chimeras (Fig. 4g). Consistently, we further demonstrated that overexpression of LPCAT1 in BMCs of SCD chimeras significantly decreased free hemoglobin compared to the controls (Fig. 4h). CBC analysis showed increased number of erythrocytes, hemoglobin concentration, and hematocrit in SCD chimeras with overexpression of LPCAT1 in BMCs (Supplementary Table 2). Erythrocyte distribution width was significantly decreased, reflecting fewer irregularly shaped cells, in SCD chimeras with overexpression of LPCAT1 in BMCs (Supplementary Table 2). Moreover, overexpressed LPCAT1 in erythrocytes of SCD chimeras significantly decreased the total number of white blood cells compared to that of SCD control chimeras (Supplementary Table 2). Similarly, circulating Leukotriene B4, prostaglandin E2 and IL-6 were also significantly decreased in SCD chimeras with the overexpression of LPCAT1 (Fig. 4i–k). Finally, histological studies revealed that the overexpression of LPCAT1 in SCD chimeras reduced splenic damage, congestion, and necrosis (Fig. 4l). Moreover, the congestion, vascular damage, and necrosis in other tissues, including lungs, livers, and spleens, were remarkably reduced in SCD chimeras with overexpression of LPCAT1 in HSCs (Fig. 4l). Semi-quantitative analysis of histological changes demonstrated that overexpression LPCAT1 in BMCs in SCD chimeras resulted in significant improvement in comparison with that in controls (Supplementary Fig. 3). Altogether, we provided strong evidence that overexpression of LPCAT1 specifically in BMCs leads to increased erythrocyte LPACT1 activity, in turn decreases LysoPC and increases PC membrane contents in the SCD mouse erythrocytes and decreases circulating AA and eventually attenuates sickling, hemolysis, inflammation and tissue damage in SCD mice.

### Lysophospatidylcholine directly promotes hypoxia-induced sickling in primary mouse erythrocytes

**Figure 5:**
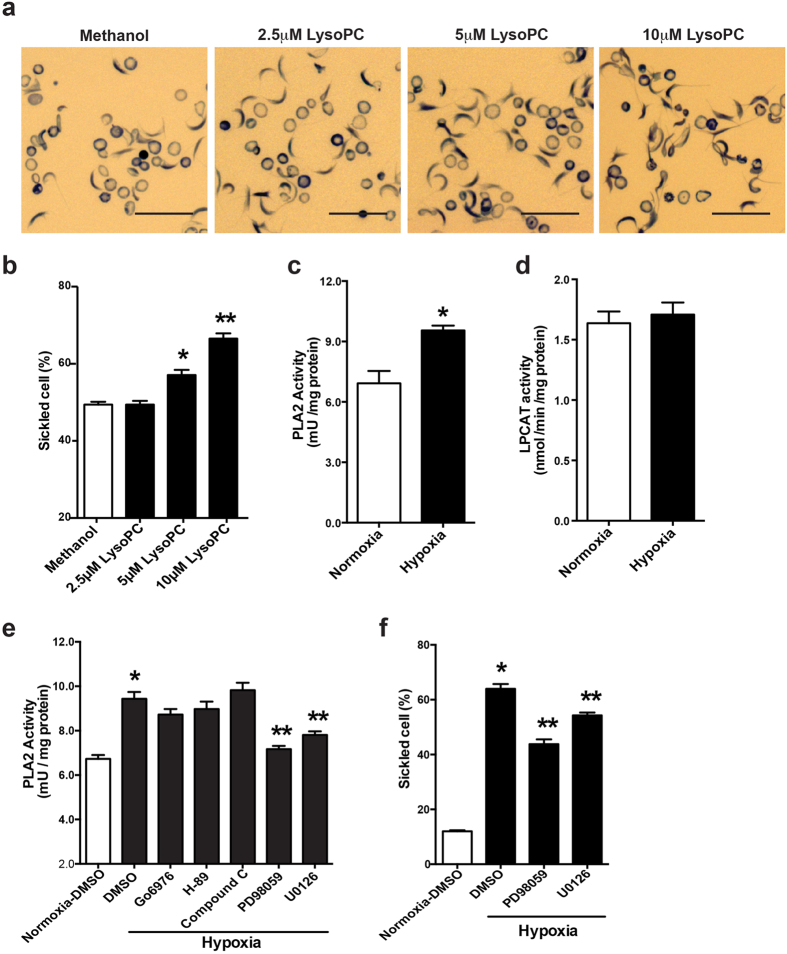
Lysophosphatidylcholine directly promotes hypoxia-induced sickling in cultured primary SCD mouse erythrocytes and hypoxia preferentially induces PLA2 but not LPCAT via MEK/ERK signaling. (**a**) Representative blood smears following treatment of blood from SCD Tg mice with Methanol (vehicle) or different concentration of LysoPC (2.5 μM, 5 μM and 10 μM as indicated) under 4% oxygen condition for 2 hours. Scale bar, 20 μm. (**b**) Quantification of (**a**). Data were represented as the mean percentage of sickle cells ± SEM. n = 3. *P < 0.05 and **P < 0.01 versus cultured SCD erythrocytes treated with DMSO or methanol. (**c,d**) PLA2 activity (**c**) and LPCAT activity (**d**) were measured in the cultured SCD mouse erythrocytes following 2 hours under normal air or 4% oxygen conditions. Error bars, SEM; n = 6 per group. *P < 0.05 versus SCD mouse erythrocytes under normoxic condition. (**e**) PLA2 activity was measured in primary cultured SCD mouse erythrocytes treated with DMSO, PKC inhibitor Go6976 (1 μM), MEK inhibitors PD98059 (20 μM) and U0126 (10 μM), PKA inhibitor H-89 (10 μM) and AMPK inhibitor compound C (10 μM) under 4% oxygen condition for 2 hours. PLA2 activity in SCD erythrocytes treated with DMSO under normoxic condition was used as a basal control. Error bars, SEM; n = 5 per group. (**f**) Quantification of blood smear analysis of cultured SCD mouse erythrocytes treated with DMSO or two different MEK inhibitors. Data are represented as the mean percentages of sickle cells ± SEM (n = 5). *P < 0.05 versus SCD mouse erythrocytes treated with DMSO under normoxia. **P < 0.05 relative to DMSO-treated group under hypoxia condition.

Increased LysoPC is reported to promote curvature of cellular shape^32^. Here we extend *in vivo* study to *in vitro* study to assess if increased LysoPC due to imbalanced Lands’ cycle directly promotes sickling. Specifically, we conducted *in vitro* studies by directly treating purified mouse sickle erythrocytes with or without LysoPC under hypoxia conditions. Consistent with *in vivo* studies, we found that LysoPC treatment significantly enhanced hypoxia-induced sickling in a dosage-dependent manner (Fig. 5a,b). Thus, we conclude that elevated LysoPC-resulting from impaired Lands’ cycle directly promotes hypoxia-induced sickling.

### Hypoxia preferentially induces PLA2 but not LPCAT activity via MEK/ERK signaling in cultured primary SCD mouse erythrocytes

SCD mice and patients constantly face hypoxia. Our findings showing that erythrocyte Lands’ cycle is imbalanced in SCD mice and that hypoxia-induced sickling was directly enhanced by LysoPC, a major product of imbalanced Lands’ cycle (Fig. 5a,b) raise an intriguing possibility that hypoxia is likely a previously unrecognized factor regulating Lands’ cycle. To test this hypothesis, we compared PLA2 and LPCAT activity under normoxia (21% O_2_) and hypoxic condition (4% O_2_) in cultured erythrocytes isolated from SCD mice. We found that hypoxia significantly induced PLA2 activity in cultured mouse sickle erythrocytes compared to normoxia (Fig. 5c). However, LPCAT activity was not affected by hypoxia treatment (Fig. 5d).

Next, we aimed to determine the specific signaling pathways involved in regulating hypoxia-induced PLA2 activity in mouse sickle erythrocytes. To address this question, we screened a series of molecules known to regulate cPLA2 phosphorylation and activation in other cell types, including protein kinase C (PKC)^33^,^34^, mitogen-activated protein kinase (MEK)^35^,^36^,^37^, protein kinase A (PKA)^38^, and AMP-activated protein kinase (AMPK)^39^. Among all of these tested candidates, we found that hypoxia-induced cPLA2 activity was significantly reduced by PD98059, a specific MEK1/2 inhibitor (Fig. 5e). However, there was no obvious effect of other kinase inhibitors on hypoxia-induced PLA2 activity (Fig. 5e). To further confirm the effect of MEK/ERK signaling cascade on PLA2 activity, we chose to treat SCD erythrocytes with U0126, another structurally different and specific MEK1/2 inhibitor. Similar as PD98059, U0126 treatment significantly decreased hypoxia-induced PLA2 activity (Fig. 5e). Moreover, analysis of blood smears showed that PD98059 and U0126 treatment significantly attenuated hypoxia-induced cell sickling (Fig. 5f). These findings revealed that MEK/ERK signaling is a key mechanism underlying hypoxia-induced PLA2 activation and subsequently sickling in mouse erythrocytes.

### Erythrocyte Lands’ cycle is impaired in SCD patients

**Figure 6:**
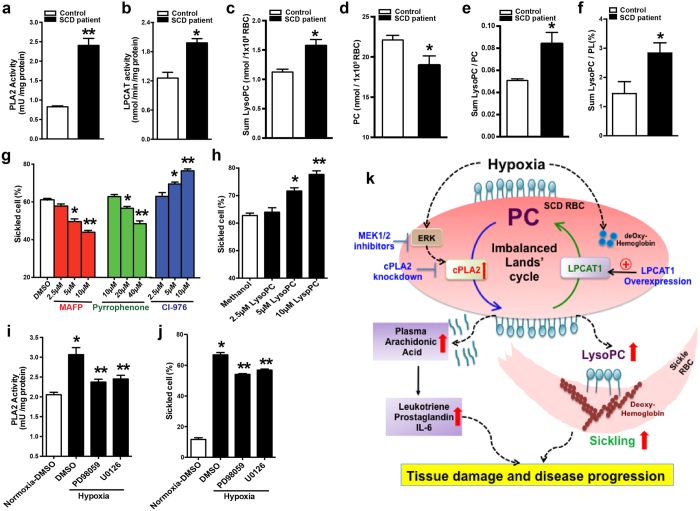
Imbalanced Lands’ cycles is seen in SCD patients and contributes to hypoxia-induced sickling by preferentially inducing PLA2 activity in MEK/ERK-dependent manner. (**a,b**) Measurement of PLA2 activity (**a**) and LPCAT activity (**b**) in the erythrocytes from healthy volunteers (control, *n* = 15) and patients with SCD (*n* = 22). (**c**–**f**) LysoPC content (**c**), PC content (**d**), the ratio of LysoPC/PC (**e**) and the percentage of LysoPC/PL (**f**) were quantified in the erythrocytes of controls and individuals with SCD. Error bars, SEM; n = 8. *P < 0.05 versus control, **P < 0.01 versus control. (**g**) Quantification of blood smear of human SCD erythrocytes treated with DMSO or different dosage of cPLA2 inhibitors, MAFP and Pyrrophenone or LPCAT inhibitor, CI-976 under 4% oxygen condition for 2 hours, respectively. (**h**) Quantification of blood smears of human SCD erythrocytes treated with methanol (vehicle) or different concentrations of LysoPC under 4% oxygen conditions for 2 hours. Error bars, SEM; n = 6. *P < 0.05, **P < 0.01 versus controls. (**i**) Measurement of PLA2 activity in human SCD erythrocytes treated with DMSO or two different MEK inhibitors, PD98059 (20 μM) and U0126 (10 μM) under hypoxic condition, respectively. (**j**) Quantification of blood smear analysis of human SCD erythrocytes treated with DMSO or two different MEK inhibitors. Data are represented as the mean percentages of sickled cells ± SEM (n = 6). *P < 0.05 versus SCD Tg mice treated with DMSO under normoxia. **P < 0.05 relative to DMSO-treated group under hypoxia condition. (**k**) Working Model: hypoxia preferentially induced elevation of activity of erythrocyte cPLA2 but not LPACT1 via MEK/ERK signaling cascade results in increased generation of erythrocyte LysoPLs and free fatty acids, in particular LysoPC and AA, and subsequently increased release of AA to the circulation in SCD. Under hypoxia, increased erythrocyte membrane LysoPC and deoxygenated HbS work together to promote sickling. Moreover, elevated circulating AA leads to increased production of multiple inflammatory mediators including leukotrienes and prostaglandins. As such, imbalanced erythrocyte Lands’ cycle-mediated elevated LysoPC and AA are pathogenic to induce sickling, inflammation and tissue damage. Thus, correcting impaired Lands’ cycle is a novel therapeutic approach for SCD management.

To translate our mouse finding to humans, we measured PLA2 and LPCAT activity, lysoPC, PC and PL levels in erythrocytes and circulating AA in patients with SCD and normal race and age-matched controls (for human subject information see Supplementary Table 3). Similar to SCD mice, we found that erythrocyte PLA2 and LPCAT activity in erythrocytes were significantly elevated up to 2.5 folds and 1.5 folds, respectively, in patients with SCD compared to controls (Fig. 6a,b). Supporting this finding, we further showed that the LysoPC level was significantly increased, while PC content was reduced in the erythrocytes from humans with SCD compared to the controls (Fig. 6c,d). As such, the ratio of LysoPC/PC and the percentage of LysoPC/PL were significantly increased in the erythrocytes isolated from individuals with SCD compared to the controls (Fig. 6e,f). These results indicated that imbalanced Lands’ cycle mediated induction of erythrocyte membrane LysoPC contents was also seen in SCD patients.

Using different inhibitors we investigated whether cPLA2 and LPCAT1 were the two major subtypes functioning in erythrocytes of SCD patients as seen in SCD mouse erythrocytes. We found that MAFP and pyrrophenone significantly inhibited PLA2 activity in cultured human sickle erythrocytes in a dosage-dependent manner (Supplementary Fig. 4a,b). In contrast, BEL showed no obvious inhibitory effect on PLA2 activity at a concentration up to 40 μM (Supplementary Fig. 4c). Similar to mouse findings, CI-976 inhibited LPCAT activity in a dosage-dependent manner (Supplementary Fig. 4d). Thus, we identified that cPLA2 and LPCAT are two major components of Lands’ cycle functioning in erythrocytes of SCD patients and that elevated LPCAT is insufficient to repair LysoPC generated by the overly active cPLA2 resulting in an imbalanced Lands’ cycle.

### Imbalanced Lands’ cycle enhances hypoxia-induced sickling in human sickle erythrocytes by inducing cLPA2 activity in a MEK/ERK dependent manner

To determine the pathophysiological significance of imbalanced Lands’ cycle in sickling in humans, we incubated erythrocytes from humans with SCD under hypoxic conditions in the presence or absence of MAFP, pyrrophenone and CI-976. Consistent with our *in vivo* mouse studies, pretreatment with MAFP and pyrrophenone significantly reduced the percentage of sickled cells under hypoxic conditions in a dosage-dependent manner (Fig. 6g). In contrast, pretreatment with CI-976 significantly enhanced sickling induced by hypoxic conditions in a dosage-dependent manner (Fig. 6g). Moreover, we validated pharmacological studies and showed that LysoPC treatment directly enhances hypoxia-induced sickling in human sickle erythrocytes (Fig. 6h).

Similar to mouse results, we found that hypoxia-mediated increased PLA2 activity was specifically blocked by two independent MEK1/2 inhibitors, PD98059 and U0126 (Fig. 6i). Similar as mouse studies, we found that both inhibitors attenuated hypoxia-induced sickling (Fig. 6j). Thus, we revealed that impaired Lands’ cycle underlies the elevation of lysoPC and contributes to hypoxia-induced sickling by inducing cPLA2 in a MEK/ERK-dependent manner in SCD patients.

## Discussion

Although Lands’ cycle was discovered nearly six decades ago, the functional role and cellular regulation of Lands’ cycle under physiological and pathological conditions are poorly understood. Using a nonbiased high-throughput metabolomic screen, we determined that an imbalanced erythrocyte Lands’ cycle contributes to sickling, a central pathogenic process of SCD in both humans and mice by promoting the accumulation of LysoPC in sickle erythrocytes. Correcting the imbalance of Lands’ cycle by knockdown of cPLA2 or overexpression of LPACT1 in HSCs of BMT SCD chimeras significantly attenuates sickling, hemolysis, inflammation and multiple tissue damage by reducing erythrocyte membrane LysoPC content and circulating AA. Mechanistically, we revealed that hypoxia preferentially inducing cPLA2 activity but not LPCAT1, a previously unrecognized causative factor underlying imbalanced Lands’ cycle in SCD erythrocytes of humans and mice. We further discovered that MEK1/ERK is a key signaling cascade responsible for hypoxia-induced cPLA2 activity in SCD erythrocytes. Overall, our studies have identified a pathological role of imbalanced erythrocyte Lands’ cycle in SCD, revealed the molecular basis regulating Lands’ cycle and immediately provided novel therapeutic possibilities for the disease.

Using high throughput metabolomic screening, we observed that erythrocyte membrane LysoPC content and circulating AA are the most elevated among all of the lipids detected in SCD mice. These findings led us to further discover that Lands’ cycle is imbalanced due to an insufficiently elevated LPCAT1, a repairing enzyme, to counteract overly active cPLA2 in both human and mouse sickle erythrocytes. Of note, early studies hinted that increased LysoPC is involved in generating curvature of cell membranes^32^ and mediating the formation of tubules connecting the Golgi stacks^40^,^41^. AA was reported to be elevated in SCD patients and is a precursor of multiple immune mediators including leukotrienes and prostaglandin^42^,^43^,^44^,^45^. However, the *in vivo* pathological significance of imbalanced Lands’ cycle-mediated increased LysoPC and AA in SCD has not been recognized prior to our studies. Using quasi-genetic studies with knockdown of cPLA2 or overexpression of LPCAT1 specifically in SCD BMT chimeras, we demonstrated that correcting the imbalance of Lands’ cycle by reducing cPLA2 activity or inducing LPCAT1 activity significantly attenuated sickling, hemolysis, inflammation and tissue damage by reducing erythrocyte membrane LysoPC, the ratio of LysoPC/PC and LysoPC/PL and circulating AA levels. Consistent with decreased circulating AA seen in SCD chimeras with specific knockdown of cPLA2 or overexpression of LPACT1, we further discovered that leukotriene and prostaglandin levels were also significantly reduced in these mice. Extending from *in vivo* mouse studies, we provide *in vitro* human and mouse evidence that LysoPC treatment directly induced sickling. Thus, both *in vitro* and *in vivo* studies support the pathological role of imbalanced Lands’ cycle in promoting hypoxia-induced sickling by increasing LysoPC/PC ratio and inflammation by inducing circulating AA and thus immediately provide innovative therapeutic targets to treat SCD (Fig. 6k).

Nothing is known about how Lands’ cycle is imbalanced in SCD erythrocytes. Intriguingly, we found that hypoxia is the key mechanism responsible for imbalanced Lands’ cycle in SCD erythrocytes by preferentially inducing cPLA2 activity but not LPCAT activity in cultured SCD mouse and human erythrocytes. These finding led us to further discover that the MEK/ERK cascade is a previously unrecognized signaling pathway involved in hypoxia-induced cPLA2 activity inside SCD erythrocytes. MEK/ERK is known to be involved in the activation of multiple signaling pathways in SCD erythrocytes including adenosine-mediated sphingosine kinase 1 (SPHK1) activation^46^ and adhesion of SCD erythrocyte to endothelium^47^,^48^,^49^. Notably, previous studies identified that elevated erythrocyte sphingosine-1-phosphate (S1P)-mediated by ERK-dependent SPHK1 activation promotes sickling^30^. Like SPHK1, here we demonstrated for the first time in both human and mouse SCD erythrocyte MEK/ERK is a key signaling cascade underlying hypoxia-induced cPLA2 activation. Similar as S1P, MEK/ERK-dependent activation of cPLA2-mediated increased LysoPC production promotes hypoxia-induced sickling. Thus, MEK/ERK is a common signaling network underlying hypoxia-induced activation of SPHK1^46^ and cPLA2 in SCD erythrocytes. Altogether, although hypoxia-induced polymerization of deoxygenated HbS has been long considered as an initial trigger to induce sickling, previously published^30^ and our current studies support a novel working model by which hypoxia-mediated alteration of erythrocyte lipid bilayer membrane components including increased S1P and LysoPC coupled with deoxygenated HbS work together to promote sickling in a MEK/ERK-dependent manner (Fig. 6k).

In conclusion, we provide both human and mouse evidence for the pathological role and regulatory mechanism of imbalanced Lands’ cycle in the process of sickling. Our findings also identified multiple therapeutic targets to treat and prevent sickling, inflammation and disease progression. Overall, our findings revealed pathogenic nature of impaired erythrocyte Lands’ cycle in SCD and immediately provide new therapeutic possibilities for multiple types of hemolytic disorders facing hypoxia.

## Methods

### Human subjects

Individuals with sickle cell disease in the steady state were identified by hematologists on the faculty of the University of Texas Medical School at Houston. Subjects participating including 15 control and 22 SCD patients in this study had no blood transfusion for at least 6 months before blood samples were collected. Control human subjects were of African descent and were free of hematological disease. Signed informed consent was obtained from all subjects. The research protocols were approved by the University of Texas Health Science Center at Houston and Brody School of Medicine of East Carolina University Committees for the Protection of Human Subjects. All experiments involved in human samples were performed in accordance with relevant guidelines and regulations. Detailed information regarding patients and controls can be found in Supplementary Table S3.

### Mice

The Berkley SCD transgenic mice (Tg [Hu-miniLCR*α1*^*G*^*γ*^*A*^*γδβ*^*S*^] *Hba*^*0*^//*Hba*^*0*^*Hbb*^*0*^//*Hbb*^*0*^) expressing exclusively human sickle hemoglobin (HbS) had been previously described^11^,^50^. The SCD Tg mice were produced by breeding homozygous males with heterozygous female mice. Four-week-old mice were genotyped by PCR. C57BL/6 mice used as controls were purchased from Harlan. Animal care was in accordance with the University of Texas Health Science Center at Houston and NIH guidelines. Research protocols were reviewed and approved by the University of Texas Health Science Center at Houston Animal Welfare Committee.

### Metabolomic profiling

Nonbiased metabolomic screening of whole blood and plasma of wild type (WT) mice and SCD mice (*n* = 6 for each group) was performed using LC/GC-MS as described previously^30^,^51^. Specifically, a Thermo Fisher linear ion-trap mass spectrometer with Fourier transform and a Mat-95 XP mass spectrometer were used to analyze 7,000 named metabolites. The LC/MS POS platform was optimized for compounds that show positive ionization as described previously^51^. There were 251 small metabolites detected in the plasma and whole blood of both the controls and SCD mice. The combinations of groups were analyzed using Welch’s 2-sample *t* test, following log transformation and imputation with minimum observed values for each compound. *P* < 0.05 was considered significant. q-value was used as a measure of “false discovery rate (FDR)”. A FDR of 0.05 means that on average 5% of the truly null features in the study will be called significant^52^.

### Arachidonic acid measurement

Measurement of arachidonic acid was performed by LIPID MAPS of the University of California, San Diego University. Mouse plasma and erythrocyte arachidonic acid (AA) was extracted and analyzed by gas chromatography mass spectrometry (GC/MS) essentially as described previously^53^,^54^. Briefly, 200 μl of plasma and 1 × 10^6^ red blood cells in PBS were mixed with a deuterated internal standard (Cayman Chemical, Ann Arbor, MI) respectively and extracted twice with 0.05 N methanolic HCl/isooctane (1:3, v/v) and the combined isooctane layers were evaporated to dryness. The extracted free fatty acids were dissolved in 1% diisopropylehylamine in acetonitrile and derivatized with 1% pentafluorobenzyl bromide. The fatty acid esters were analyzed by GC/MS on an Agilent 6890N gas chromatograph equipped with an Agilent 5973 mass selective detector (Agilent, Santa Clara, CA). AA quantitation was achieved by the stable isotope dilution method^55^. A standard curve was generated by linear regression analysis of the ratio between primary standard peak area and internal standard peak area plotted versus the amount of primary standard.

### Blood collection, complete blood count and treatment of human and mouse erythrocytes *in vitro*

Blood was collected from humans and mice as previously described^30^,^46^. Briefly, approximately 7 ml blood was withdrawn from forearm veins of normal individuals and SCD patients. 3 ml blood was collected in three 1.5 ml tubes containing sodium heparin and mixed well. Packed erythrocytes were stored at −80 °C for phospholipid isolation and quantification, and for PLA2 and LPCAT activity measurement (see below). An additional 4 ml of blood was collected with EDTA as an anti-coagulant and used for morphological study, complete blood count (CBC) and hemoglobin electrophoresis. Mouse blood was collected similar to human blood as described above except for smaller volumes.

### Mature erythrocytes and reticulocytes isolation from SCD mice

Erythrocytes and reticulocytes were separated by centrifugation on a 2-step gradient of isotonic Percoll as previously described^56^. Firstly, 90% Percoll was made by mixing 90 ml of Percoll plus (GE Healthcare) with 10 mL of 9% sodium chloride. Then, 70% or 75% Percoll was prepared by dilution of 90% Percoll with different amounts of 0.9% sodium chloride, respectively. The upper layer (2 mL) of the gradient was a 70% Percoll solution, whereas the lower layer (2 mL) was a 75% Percoll solution. 200 μL of mouse sodium heparin anti-coagulated blood was very gently layered on the top of the 70% Percoll. The sample was centrifuged at 1000 g for 20 minutes at room temperature in a centrifuge with swinging tube holder. After centrifugation, the top layer (platelets and white blood cells) was removed and the middle layer (reticulocytes and granulocytes) and bottom layer (mature erythrocytes) were collected into different 1.5-mL tubes respectively and washed twice with PBS. The reticulocyte fraction was resuspended in 0.5 mL PBS with the addition of 20 μg anti-granulocyte antibody (BD Pharmingen) and incubated at room temperature for 30 minutes. The cell suspension was washed once by PBS, followed by addition of anti-rat IgG-magnetic beads and incubation at room temperature for 30 minutes. Antibody-bound cells (granulocytes) were pulled down by magnetic bar (Bio-Rad). The supernatant (reticulocytes) was centrifuged at 1000 g for 5 minutes at room temperature. Cell pellets were washed twice with PBS, frozen in liquid nitrogen and stored at −80 °C for PLA2 and LPCAT activity assay.

### Erythrocyte membrane lipid extraction, thin-layer chromatographic analysis and phosphate measurement

Extraction of erythrocyte membrane lipid was performed as previously described^57^,^58^. 1 × 10^9^ erythrocyte pellet was re-suspended in 0.3 ml of 0.5 N NaCl in 0.1 N HCl. Then 0.9 ml of chloroform-methanol (1:2) was added to cell lysate and the mixed solution was vortexed for 30 min. Then 0.3 ml of 0.5 N NaCl in 0.1 N HCl was added to the solution and continued to vortex for another 10 min. Afterwards, the mixture was centrifuged for 5 min in an Eppendorf Table Centrifuge. The upper water-methanol phase was discarded, and lower chloroform lipid phase was carefully transferred to a new Eppendorf tube. Lipid phase from each sample was evaporated to dryness under nitrogen and then re-dissolved in a small volume (50–100 μl) of chloroform. Re-suspended lipid extracts were subjected to thin-layer chromatography (TLC) using a HPTLC 60 plate (EMD) developed with solvent consisting of chloroform/methanol/acetic acid [65:25:10 (vol/vol)] described by Tan *et al*.^57^. After drying the plate in the hood, the individual lipid components were examined by staining with iodine vapor. When spots were visualized, all spots were scraped from the plate and transferred to new tubes. The individual phospholipid was measured by quantification of inorganic phosphorus using malachite green as described previously^59^,^60^,^61^.

### Isolation of total erythrocytes and treatment of human and mouse erythrocytes *in vitro*

Collected blood was centrifuged at 240 g for 10 min at room temperature, followed by aspiration of plasma and white interface. RBCs were washed twice with Ham’s F-10 nutrient mix (ThermoFisher Scientific) and then resuspended with Ham’s F-10 nutrient mix. RBCs were then incubated with or without PLA2 inhibitors including Methyl Arachidonyl Fluorophosphonate (MAFP) (Cayman Europe), pyrrophenone, bromoenol lactone (BEL) (Cayman Europe) or an LPCAT inhibitor CI-976 (Cayman Europe) under ambient oxygen (normoxia) or different levels of hypoxia (for 3 hours with shaking at 37 °C). At the end of experiments, PLA2 and LPCAT enzyme activity were measured and the percentage of sickled cells was determined as described previously^30^.

### Measurement of PLA2 activity in the erythrocytes

Pelleted red blood cells were lysed in a buffer containing 50 mM Tris-HCl, pH 8.9, 100 mM NaCl, 1 mM Na-orthovanadate. The total protein concentration was measured with Pierce™ BCA Protein Assay Kit (Life Technologies) and PLA2 activity was measured by EnzChek Phospholipase A2 Assay Kit (Life Technologies).

### Measurement of LPCAT activity and protein level in the erythrocytes

Erythrocyte LPCAT activity was determined by measuring the formation of [^14^C] PC from lysoPC and [^14^C] acyl-CoA^26^. All reactions were performed in 100 mM Tris-HCl, pH 7.4 containing 1 μM CaCl2, 0.015% Tween-20, 200 μM lysoPC (Avanti Polar Lipids), and 20 μM [1-^14^C] oleoyl-CoA (0.01 μCi) (Perkin Elmer) at 37 °C with isolated erythrocyte membrane protein in a total volume of 200 μl. The reaction was terminated by adding 800 μl of chloroform/methanol (2:1, vol/vol) to the incubation mixture. Then lipids were extracted and then resolved by Thin-layer chromatograph (TLC) silica plates with chloroform/methanol/acetic acid/0.9% NaCl (100:50:16:5, vol/vol). TLC plates were then exposed to phosphor-imaging screening (Bio-Rad) and scanned for radioactive signals as indications of the formation of PC. A part of membrane protein was used to detect LPCAT1 protein expression by Western blot. Approximately 20 μg membrane protein was run on 10% SDS-PAGE gels. After protein was transferred to PVDF membrane, the membrane was blocked with 5% nonfat milk and incubated with anti-LPCAT1 antibody (ab94903, Abcam) and Donkey anti-Rabbit secondary antibody (Santa Cruz Biotechnology Inc.), respectively.

### Lentivirus production and bone marrow transduction

Recombinant lentivirus particles were produced in HEK293T cells by transient transfection of lentivirus packaging plasmids, including 4 plasmids expressing Gag-Pol (pHDM-Hgpm2), Tat (pHDM-tat1b), Rev (pRC-CMV-rev1B), and VSV-G (pHDM VSV-G), and a pool of 3 recombinant lentiviral vectors, each encoding shRNA specific for cPLA2 (Santa Cruz Biotechnology Inc., sc-35098-SH) or scrambled shRNA (Santa Cruz Biotechnology Inc., sc-108060). Lentiviral particles packaged with LPCAT1 overexpressing plasmid were produced by co-transfection of lentivirus packaging plasmids, as described above, with pHAGE-EF1α-3xFlag vector or pHAGE-EF1α-3xFlag-LPCAT1 into HEK293T cells. For construction of pHAGE-EF1α-3xFlag-LPCAT1 plasmid, full length of LPCAT1 coding sequence was inserted into pHAGE-EF1α-3xFlag vector to generate pHAGE-EF1α-3xFlag-LPCAT1 plasmid. Cell culture media containing viruses was collected 3 times within 72 hours after transfection and concentrated by centrifugation^62^. Concentrated virus stocks had a titer greater than 2 × 10^8^ per ml and were stored at −80 °C before bone marrow cell (BMC) transduction. BMCs isolation and viral transductions were performed by previously described^30^. The efficiency of lentivirus transduction was assessed by detection of GFP expression in BMCs using flow cytometry.

### Irradiation and bone marrow transplantation

C57BL/6 mice (12 to 14 weeks of age) were treated with neomycin at 2 μg/ml in drinking water the day before irradiation as described previously^63^. Next day, mice were exposed to 5 Gy body irradiation with a gamma irradiator (Gammacell, MDS Nordion). Four hours later, the mice were exposed to the same dose of irradiation. BMCs transduced by recombinant lentivirus as described above were injected into the *tail veins* of irradiated mice (1 × 10^6^ BMCs per mouse). After BMT, the mice were injected intraperitoneally with 1 μg/kg body weight erythropoietin per day for 3 days and treated with 2 μg/ml neomycin in drinking water for 2 weeks. Mice were sacrificed 16 weeks later for experiments.

### Analysis of chimerism by monitoring the percentage of hemoglobin A and hemoglobin S by High-Performance Liquid Chromatography (HPLC) in SCD chimeras

Analysis of different hemoglobin variants was performed as previously described^30^. Briefly, isolated red blood cells were fully lysed by addition of three volumes of water. A 20 μl of cell lysate was added to 180 μl of mobile phase B buffer (40 mM Bis-Tris, 2 mM KCN, 0.2 M NaCl, pH 6.8), and from this stock, a 25 μl aliquot was injected into HPLC and monitored at 415 nm. Obtained peak areas were used for the quantitation of individual hemoglobin peaks. For determination of peak positions in HPLC profile, purified hemoglobin A and/or hemoglobin S were analyzed.

### Morphology study of erythrocytes

Cultured human or mice erythrocytes were fixed with 1% glutaraldehyde. Then blood smears were performed using fixed samples as described previously^12^. Blood smears stained for sickled cells were observed using an Olympus BX60 microscope. Areas without overlapped red blood cells were randomly picked, and then at least 10 fields were monitored and more than 1000 red blood cells including sickle cells were counted. The percentages of sickled cells in total counted erythrocytes were calculated.

### Plasma leukotriene B4 and prostaglandin E2 measurement

Plasma leukotriene B4 and prostaglandin E2 were measured by using ELISA kits following instructions provided by the vendor (BioAssay Systems, Hayword, CA, ADI-901-068 and ADI-900-001). Briefly, Plasma samples were firstly acidified by addition of 2 M HCl to pH of 3.5 and incubated at 4 °C for 15 minutes. Then samples were centrifuged for 2 minutes to remove any precipitates. Supernatant was loaded into pre-washed C18 reverse phase column. After a series of column washing by water, 15% ethanol and hexane, the sample was eluted from the column by addition of ethyl acetate. After evaporating samples under nitrogen, dried samples were dissolved with assay buffer and quantified using leukotriene B4 and prostaglandin E2 ELISA kit respectively.

### Mouse organ isolation and histological analysis

Mice were anesthetized and spleen, livers and kidneys were isolated. Each organ was fixed with 10% paraformaldehyde in PBS and dehydrated through graded ethanol washes, and embedded in paraffin. 5 μm sections were collected on slides and stained with hematoxylin and eosin (H&E). The semi-quantitative analysis of histological changes was conducted as previously described using a computerized program^30^,^64^. 10 digital images were taken from each H&E stained mouse tissue section at 20X magnification from different areas. The congestion, necrosis or cysts on sections were identified according to their structure and color. Briefly, the dark red color was chosen for quantification of congestion and it was performed on 10 fields/mouse tissue sections at 20X magnification using software analysis (Image Pro Plus 4.0; Media Cybernetics, Bethesda, MD, USA) Additionally, the areas of necrosis in the livers and congestion in the spleen and lung were first manually marked by using a magical pen tool available in Adobe Photoshop Program. Then the quantification was conducted on 10 fields/mouse tissue sections at 20x using software analysis (Image Pro Plus 4.0; Media Cybernetics, Bethesda, MD, USA). The whole areas of each image were considered as 100%. The percentage of pathological areas to whole area of image was recorded.

### Hemolysis analysis

The free hemoglobin in mouse plasma was quantified by using ELISA kits following instructions provided by the vendor (BioAssay Systems, Hayword, CA).

### Statistical Analysis

All data are expressed as the mean ± SEM. Data were analyzed for statistical significance using GraphPad Prism 5 software (GraphPad Software). Two-tailed Student’s *t* tests (paired or unpaired as appropriate) were applied in 2-group analysis. Differences between the means of multiple groups were compared by 1-way analysis of variance, followed by a Tukey’s multiple comparisons test. A *P* value of less than 0.05 was considered significant.

## Additional Information

**How to cite this article**: Wu, H. *et al*. Hypoxia-mediated impaired erythrocyte Lands’ Cycle is pathogenic for sickle cell disease. *Sci. Rep.* **6**, 29637; doi: 10.1038/srep29637 (2016).

## Supplementary Material

Supplementary Information
