# Supplementary material for: Hypoxia-mediated impaired erythrocyte Lands’ Cycle is pathogenic for sickle cell disease
Source: Sci Rep. 2016 Jul 20;6:29637. doi: 10.1038/srep29637 (PMC4951653; doi:10.1038/srep29637)
Supplement: Supplementary Information [file srep29637-s1.pdf]

Supplementary information

For

Hypoxia-mediated impaired erythrocyte Lands' Cycle is pathogenic for sickle cell disease

by

Hongyu Wu[1], Mikhail Bogdanov[1], Yujin Zhang[1], Kaiqi Sun[1&2], Shushan Zhao[1], Anren Song[1], Renna Luo[1], Nicholas F. Parchim[1&2], Hong Liu[1&2], Aji Huang[1], Morayo G, Adebiyi[1&2], Jianping Jin[1], Danny C. Alexander[3], Michael V. Milburn[3], Modupe Idowu[4], Harinder S. Juneja[4], Rodney E. Kellems[1&2], William Dowhan[1], Yang Xia[1&2*]

[1]Department of Biochemistry and Molecular Biology, University of Texas-Medical School, Houston, TX, USA; [2]Graduate School of Biomedical Sciences, University of Texas, Houston, TX, USA; [3]Metabolon, Inc., Durham, NC, USA; [4]Department of Internal Medicine, University of Texas-Medical School, Houston, TX, USA;

Running Title: Imbalanced Lands' cycle in sickle cell disease

Keywords: Lands' cycle; lysophosphatidylcholine; PLA2; LPCAT1; sickling; sickle cell disease

There are no conflicts of interest for any of the authors.

***Correspondence:** Yang Xia, Department of Biochemistry & Molecular Biology, University of Texas Medical School at Houston. Email: yang.xia@uth.tmc.edu

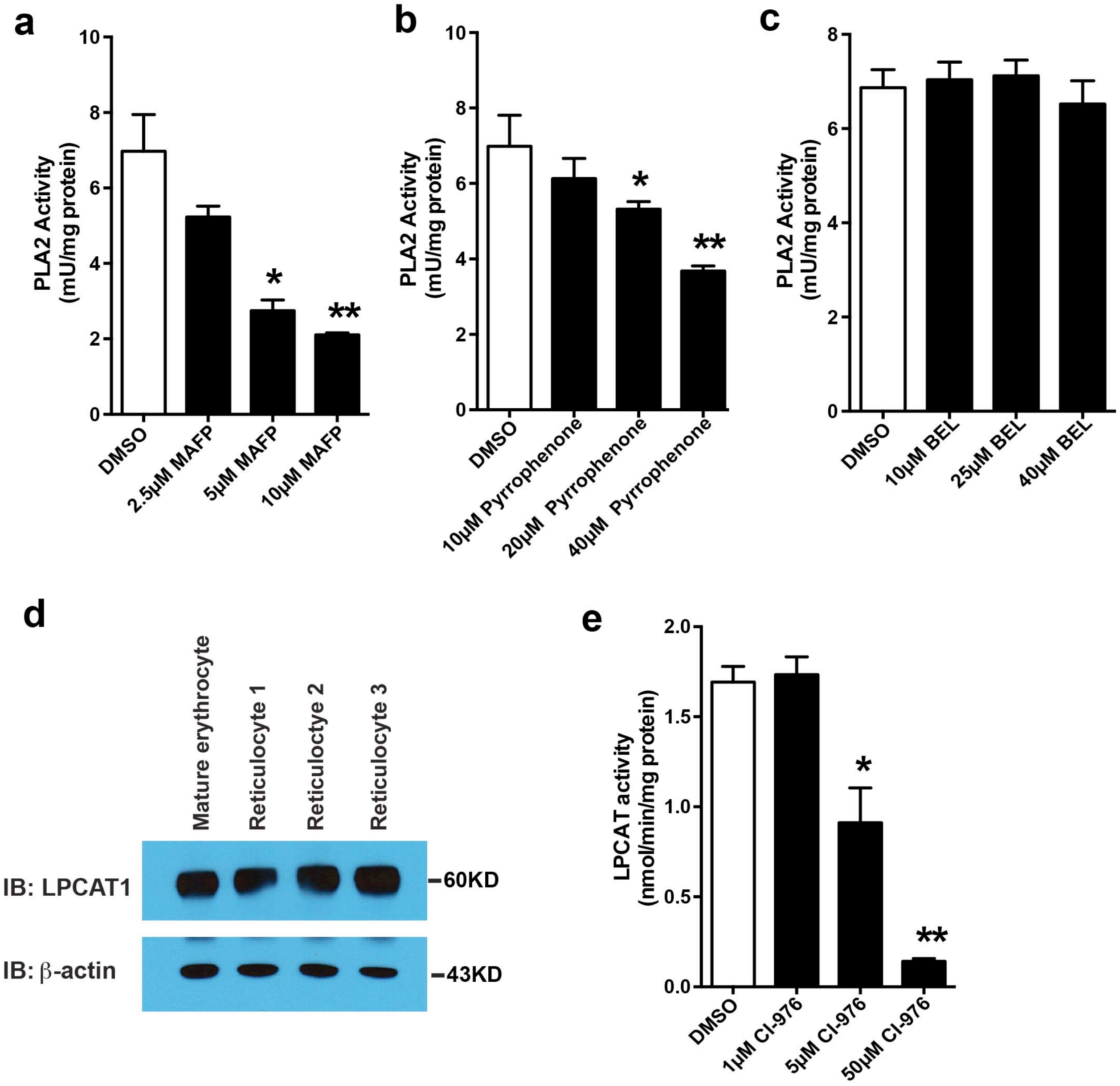

Supplementary Figure 1. Cytosolic PLA2 and LPCAT1 are the major components of Lands' cycle for PC generation in mouse SCD erythrocytes. (**a-c**) Quantification of PLA2 activity in cultured SCD Tg mice erythrocytes treated with DMSO or different dosages of cPLA2 inhibitors, MAFP (2.5μM, 5μM and 10μM) (**a**), Pyrrophenone (10μM, 25μM and 40μM) (**b**) or iPLA2 inhibitor BEL (10μM, 25μM and 40μM) (**c**) under 4% oxygen condition for 2 hours. Data are expressed as Mean± SEM; n = 5 in each group. (**d**) LPCAT protein level was measured in isolated mature erythrocytes and reticulocytes from SCD mice by Western blot. (**e**) LPCAT activity was measured in the cultured SCD mouse erythrocytes treated with different dosages of LPCAT inhibitor, CI-976 (1μM, 5μM and 50μM as indicated). Data are expressed as Mean± SEM; n = 4 in each group. *$P < 0.05$ and **$P < 0.01$ versus cultured SCD erythrocytes treated with DMSO or treated with a lower concentration of drugs,

respectively.

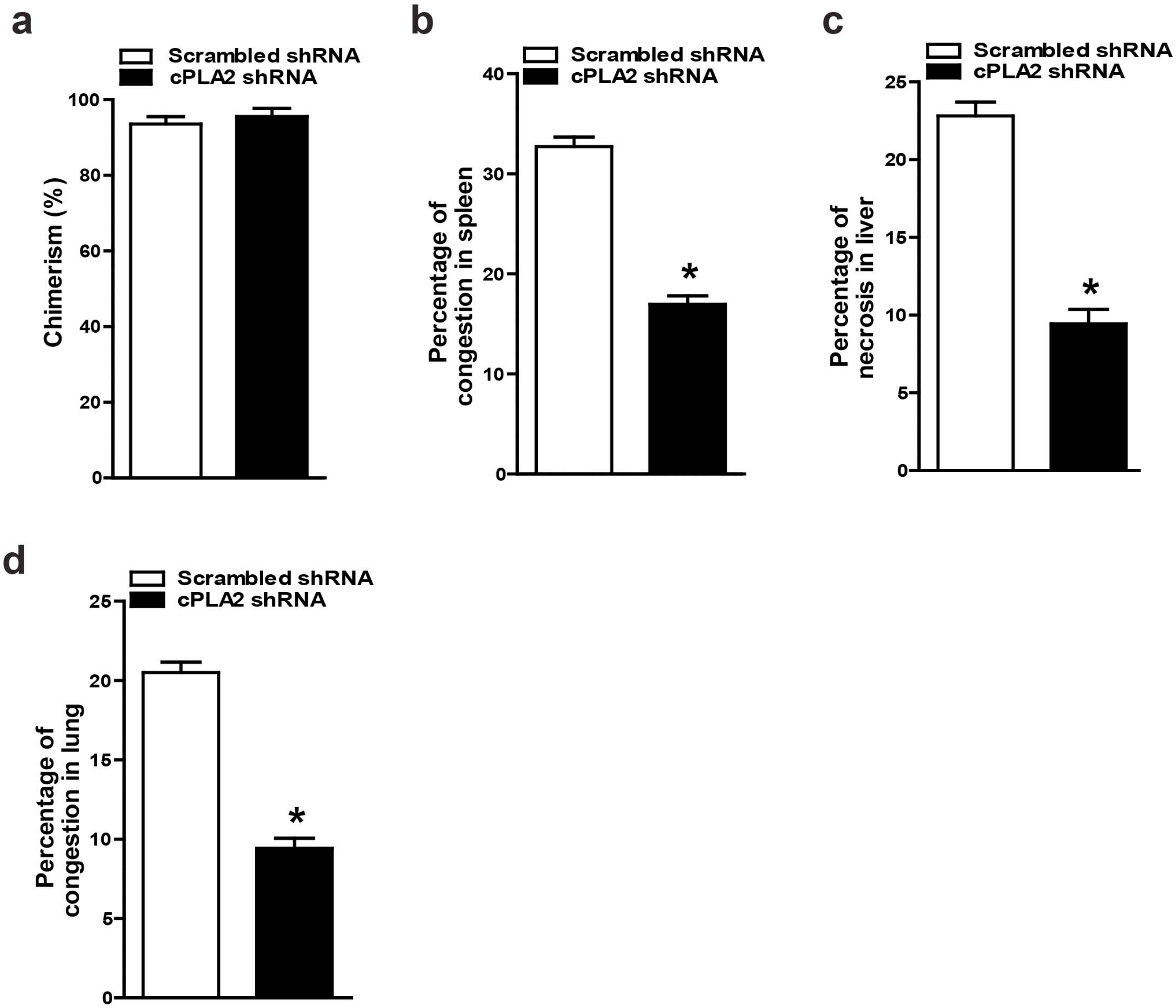

Supplementary Figure 2. Semi-quantitative analysis of tissue damage in BMT SCD chimeras with lentiviral shRNA specific knockdown of cPLA2 in hematopoietic stem cells (HSCs) and with scrambled shRNA. Related to Figure 3. (**a**) The percentage of SCD chimeras was assessed by quantification of HbS and mouse normal Hb (HbA) in erythrocytes from BMT SCD chimeras with HSC-specific knockdown of cPLA2. Data shown represent the Mean ± SEM. Data shown represent the Mean ± SEM (n = 7). (**b-d**) Semi-quantitative analysis of histological changes in spleen (**b**), liver (**c**)**,** and lung (**d**) of SCD chimeras with cPLA2 knockdown in HSCs using Image-Pro Plus software. Data are expressed as Mean± SEM; n = 5-7 in each group. *$P < 0.05$ versus SCD chimeras with BMCs infected with recombinant lentivirus encoding scrambled shRNA.

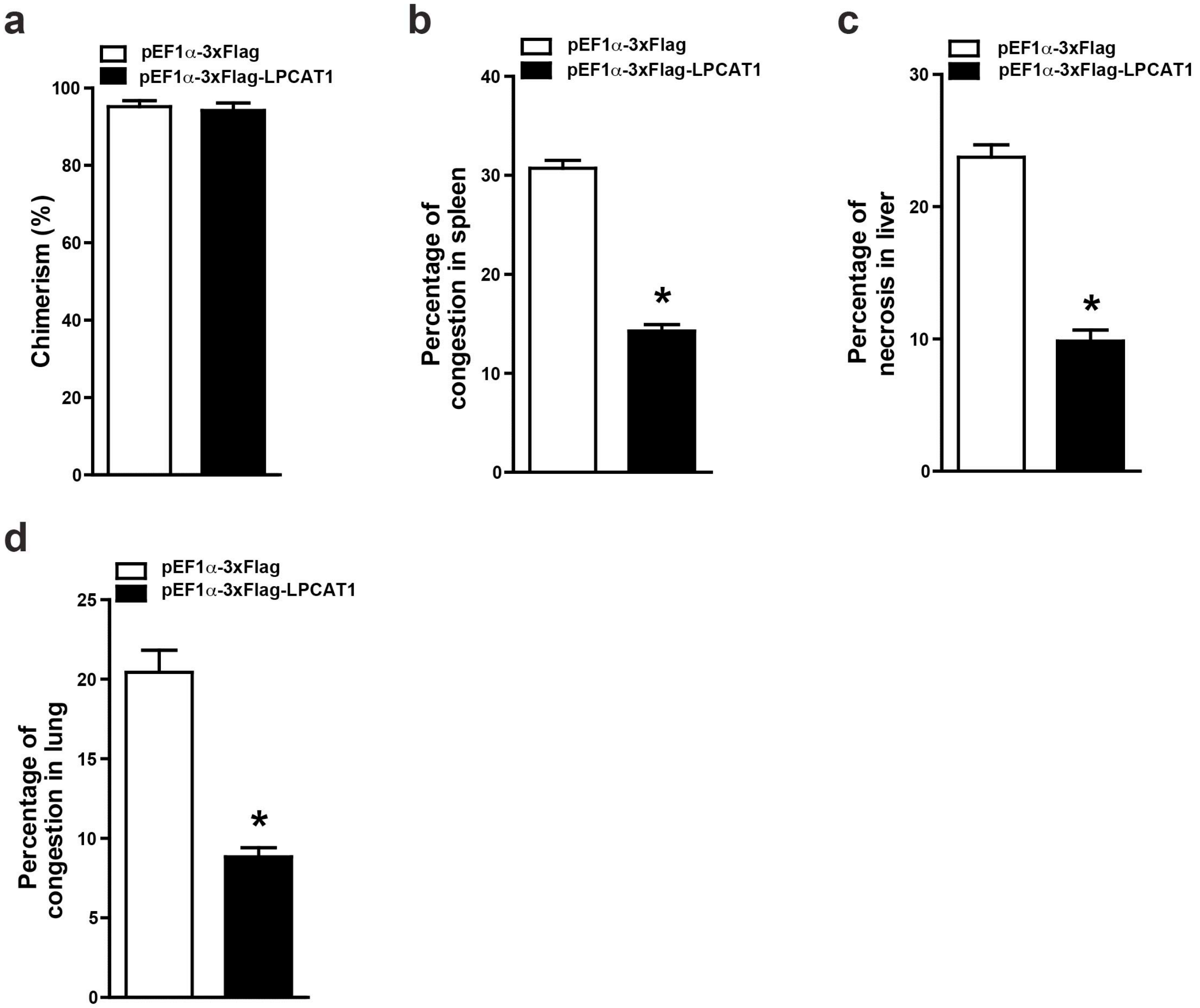

Supplementary Figure 3. Semi-quantitative analysis of tissue damage in BMT SCD chimeras with lentiviral overexpression of LPCAT1 and with control vector in hematopoietic stem cells (HSCs). Related to Figure 4. (a) The percentage of SCD chimeras was assessed by quantification of HbS and mouse normal Hb (HbA) in erythrocytes from BMT SCD chimeras with overexpression of LPCAT1. Data shown represent the Mean ± SEM. (**b-d**) Semi-quantitative analysis of histological changes in spleen (**b**), liver (**c**), and lung (**d**) of SCD chimeras with LPCAT1 overexpression using Image-Pro Plus software. Data are expressed as Mean± SEM; n = 6 in each group. $^*P < 0.05$ versus SCD chimeras with BMCs infected with recombinant lentivirus encoding control vector.

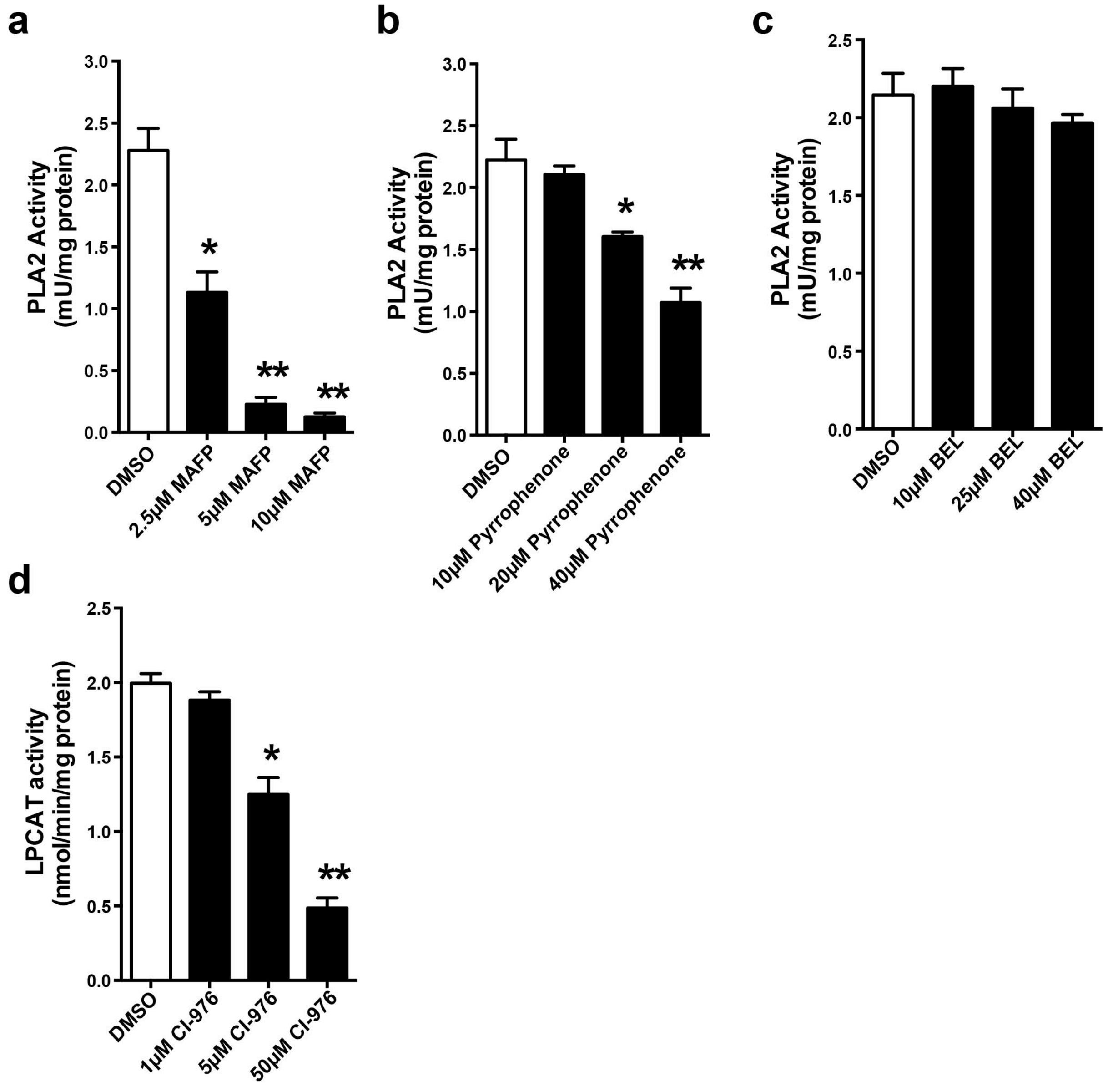

Supplementary Figure 4. Cytosolic PLA2 and LPCAT function as major components of Lands' cycle for PC formation in human SCD erythrocytes, Related to Figure 6. (**a-c**) Quantification of PLA2 activity in isolated erythrocytes from individuals with SCD treated with DMSO or different dosages of cPLA2 inhibitors, MAFP (2.5μM, 5μM and 10μM) (**a**), Pyrrophenone (10μM, 25μM and 40μM) (**b**) or iPLA2 inhibitor BEL (10μM, 25μM and 40μM) (**c**) under hypoxic condition (4%). Data are expressed as Mean± SEM; n = 5 in each group. (**d**) LPCAT activity was measured in the cultured human SCD erythrocytes treated with different dosage of LPCAT inhibitor, CI-976 (1μM, 5μM and 50μM). Data are expressed as Mean± SEM; n = 5 in each group. *$P < 0.05$ and **$P < 0.01$ versus cultured SCD human erythrocytes treated with DMSO or treated with lower concentration of drugs, respectively.

Supplementary Table 1 Hematological parameters of SCD chimeras with or without HSCs-specific cPLA2 knockdown

Mice	RBC ($X10^6$/ml)	Hb (g/dl)	HCT (%)	MCV (fl)	MCH (pg)	MCHC (g/dl)	RDW (%)	WBC ($X10^3$/ml)
Scrambled shRNA (n=7)	5.70±0.14	6.04±1.67	30.34±1.88	50.73±0.50	11.14±0.89	21.94±0.12	24.7±0.23	18.47±1.77
cPLA2 shRNA (n=5)	6.52±0.22*	8.95±0.23*	34.53±1.51*	52.9±0.45	13.77±0.05*	23.65±0.46	20.76±0.43*	9.82±0.92*

Control vector: C57BL/6 mice were transplanted with SCD Tg mice bone marrow cells transduced with recombinant lentiviral vectors encoding scrambled-shRNA sequence;
cPLA2-shRNA: C57BL/6 mice were transplanted with SCD Tg mouse bone marrow transduced with recombinant lentiviral vectors encoding shRNA specifically for cPLA2 mRNA.

SCD, Sickle cell disease transgenic mice; RBC, red blood cell, Hb, hemoglobin; HCT, hematocrit; MCV, mean corpuscular volume; MCH, mean corpuscular hemoglobin; MCHC, mean corpuscular hemoglobin concentration; RDW, red blood cell distribution width; WBC, white blood cell. *$P < 0.05$ versus untreated SCD transgenic mice.

Supplementary Table 2 Complete blood count in SCD chimeras with or without overexpression of LPCAT1 in donor bone marrow cells

Mice	RBC ($X10^6$ /ml)	Hb (g/dl)	HCT (%)	MCV (fl)	MCH (pg)	MCHC (g/dl)	RDW (%)	WBC ($X10^3$ /ml)
pEF1α-3xflag (n=6)	6.04±0.09	6.18±0.10	30.90±0.63	50.38±0.26	11.46±0.07	20.00±0.13	23.48±0.14	17.14±0.64
pEF1α-3xflag-LPCAT1 (n=6)	6.71±0.16*	8.73±0.28*	35.36±1.12*	52.50±0.88	14.04±0.10*	23.88±0.22*	20.83±0.18*	10.20±0.55*

Control vector: C57BL/6 mice were transplanted with SCD Tg mice bone marrow cells transduced with recombinant lentiviral vectors encoding control vector;
Flag-LPCAT: C57BL/6 mice were transplanted with SCD Tg mouse bone marrow transduced with recombinant lentiviral vectors encoding Flag-labeled LPCAT1 sequence.

Hb, hemoglobin; HCT, hematocrit; MCV, mean corpuscular volume; MCH, mean corpuscular hemoglobin; MCHC, mean corpuscular hemoglobin concentration; RDW, red blood cell distribution width; WBC, white blood cell. *$P < 0.05$ versus untreated SCD Tg mice.

Supplementary Table 3: Clinical information for control individuals and SCD patients

	Control	SCD patient
Number	15	22
Gender	M=5 F=10	M=10 F=12
Age (Years)	39.52±11.21	31.82±10.88
RBCs (10^6/μl)	4.73±0.32	2.41±0.50*
Hb (g/dl)	13.76±1.51	8.26±1.47*
HCT (%)	41.34±1.63	24.71±1.40*
WBC (10^3/μl)	5.64±2.01	11.66±4.05*
HbS	NT	81.74±8.30
HbA	NT	0.87±2.89
HbA2	NT	3.12±0.62
HbF	NT	14.28±8.35
Hydroxyurea	No	13

Control: Healthy volunteers; SCD patient: sickle cell disease patient; RBC: red blood cells; Hb: hemoglobin; HCT: hematocrit; WBC: white blood cell; HbS: hemoglobin S; HbA: hemoglobin A; HbA2: hemoglobin A2; HbF: hemoglobin F; NT: not tested. **P*≤0.05 vs. control. All subjects are of African-American decent.
